# Topical Delivery of Tofacitinib in Dermatology: The Promise of a Novel Therapeutic Class Using Biodegradable Dendritic Polyglycerol Sulfates

**DOI:** 10.3390/ph17010077

**Published:** 2024-01-08

**Authors:** Fatemeh Zabihi, Mariam Cherri, Xiao Guo, Fiorenza Rancan, Fabian Schumacher, Ehsan Mohammadifar, Burkhard Kleuser, Wolfgang Bäumer, Michael Schirner, Annika Vogt, Rainer Haag

**Affiliations:** 1 Institut für Chemie und Biochemie, Freie Universität Berlin, Takustr. 3, 14195 Berlin, Germany; fatemeh.zabihi@bfr.bund.de (F.Z.); mcherri6@zedat.fu-berlin.de (M.C.); ehsan@zedat.fu-berlin.de (E.M.); michael.schirner@fu-berlin.de (M.S.); 2 Clinical Research Center for Hair and Skin Science, Department of Dermatology, Venereology, and Allergy, Charité Universitaetsmedizin, 10117 Berlin, Germany; xiao.guo@charite.de (X.G.); fiorenza.rancan@charite.de (F.R.); 3 Institute of Pharmacy (Pharmacology and Toxicology), Freie Universität Berlin, Königin-Luise-Straße 2+4, 14195 Berlin, Germany; fabian.schumacher@fu-berlin.de (F.S.); burkhard.kleuser@fu-berlin.de (B.K.); 4 Core Facility BioSupraMol PharmaMS, Institute of Pharmacy, Freie Universität Berlin, Königin-Luise-Straße 2+4, 14195 Berlin, Germany; 5 Institute of Pharmacology and Toxicology, School of Veterinary Medicine, Freie Universität Berlin, Koserstr. 20, 14195 Berlin, Germany; wolfgang.baeumer@fu-berlin.de

**Keywords:** inflammatory skin disease, JAK-STAT, tofacitinib, ex-vivo skin model, dPGS-PCL, interfollicular delivery, nanoparticles, squalene

## Abstract

Inflammatory skin diseases, such as psoriasis, atopic dermatitis, and alopecia areata, occur when the regulatory tolerance of the innate immune system is disrupted, resulting in the activation of the Janus kinase-signal transducer and activator of transcription (JAK-STAT) inflammatory signaling pathway by interleukin 6 (IL-6) and other key inflammatory cytokines. JAK inhibitors, such as tofacitinib, bind to these enzymes which are coupled to receptors on cell surfaces and block the transcription of inflammatory cytokine-induced genes. The first topical applications are being marketed, yet insufficient effects regarding indications, such as alopecia areata, suggest that improved delivery technologies could help increase the efficacy. In this study, we used sulfated dendritic polyglycerol with caprolactone segments integrated in its backbone (dPGS-PCL), with a molecular weight of 54 kDa, as a degradable carrier to load and solubilize the hydrophobic drug tofacitinib (TFB). TFB loaded in dPGS-PCL (dPGS-PCL@TFB), at a 11 w/w% loading capacity in aqueous solution, showed in an ex-vivo human skin model better penetration than free TFB in a 30:70 (*v*/*v*) ethanol/water mixture. We also investigated the anti-inflammatory efficacy of dPGS-PCL@TFB (0.5 w/w%), dPGS-PCL, and free TFB in the water/ethanol mixture by measuring their effects on IL-6 and IL-8 release, and STAT3 and STAT5 activation in ex vivo skin models of simulated inflamed human skin. Our results suggest that dPGS-PCL@TFB reduces the activation of STAT3 and STAT5 by increasing the penetration of the tofacitinib. However, no statistically significant differences with respect to the inhibition of IL-6 and IL-8 were observed in this short incubation time.

## 1. Introduction

Inflammation is a physiological response of the immune system for the purpose of defending the body against infection from pathogens and other external stimulus factors [1]. Diverse pathogenic mechanisms contribute to this process, which involves the hyperactivation of immune cells or structural tissue cells [1,2]. Chronic inflammatory diseases comprise skin inflammatory disorders such as psoriasis, atopic dermatitis, and alopecia areata [3,4,5]. Activated dendritic cells (DCs) and T cells play essential roles in the signaling pathways for psoriatic inflammation and other chronic inflammation [5,6]. Specifically, cytokines derived from DCs or T cells, once activated, trigger the overabundant release of inflammatory mediators from epidermal cells [7]. Activated DCs also release interleukins, such as IL-23 and IL-12, that stimulate three populations of T cells: Th17, Th22, and Th1. These activated T cells then synthesize IL-17, causing the uncontrolled activation of keratinocytes, which in turn triggers the production of cytokines and chemokines [2,8]. These and other inflammatory mediators then bind to specific receptors on cell surfaces and regulate immune responses [9].

The onset of skin inflammation involves several steps. In the initial stage of skin inflammation, damaged cells express inflammatory mediators, such as interleukin 6 (IL-6), which can promote the differentiation or proliferation of DCs, T cells, and non-immune cells [10,11]. The upregulation of Th17 and CD4^+^ T cells then disrupts immunological tolerance and drives chronic inflammation. In this process, IL-6 binds to cell surfaces via the IL-6 transmembrane receptor (IL-6R) or soluble IL-6R (sIL-6R) [12]. The activated IL-6R complex drives the dimerization of glycoprotein 130 (gp130) inside the cell. The dimerization of gp130 in the cell cytoplasm then triggers the inflammatory signaling pathway known as the Janus kinase-signal transducer and activator of transcription (JAK-STAT) [10,13]. The JAK family of enzymes includes JAK1, JAK2, JAK3, and TYK2; the STAT family contains STAT1, STAT2, STAT3, STAT4, STAT5a, STAT5b, and STAT6 [14]. By this process, external stimulus factors, such as infections or tissue damage, promote the increased expression of IL-6, thereby inducing the alarm signals that trigger the JAK-STAT or JAK-SHP-2-mitogen-activated protein (MAP) kinase pathways. Studies show that cytokines, such as IL-6, that rely on JAK for their signal transduction are major contributors to chronic inflammation in psoriasis and atopic dermatitis. Targeting the IL-6 signal pathway may therefore be beneficial in treating such diseases [11,15].

JAK enzyme inhibitors have proven to be an efficient therapeutic approach for several inflammatory and autoimmune diseases, including rheumatoid arthritis, psoriasis, and atopic dermatitis [14,16,17,18]. Ruxolitinib, tofacitinib, baricitinib, and oclacitinib are the first generation of JAK inhibitors [19,20]. Tofacitinib was the first one to be approved by the Food and Drug Administration (FDA), and the European Medicines Agency (EMA) for treating rheumatoid arthritis and other immune-mediated diseases [21]. Tofacitinib specifically inhibits JAK1 and JAK3 and the phosphorylation of STAT5, STAT1, and STAT3 in response to IL-6 or IL-15 exposure [20,21]. Various immune-mediated skin diseases are also tied to cytokines and their stimulation of JAK-STAT signaling pathways. Topical administration of JAK inhibitors for treating skin disease is favorable over oral application due to the less severe side effects and the enhanced therapeutic concentration of the drugs at the target site [22,23]. Tofacitinib was among the first candidates of JAK inhibitors to be introduced for dermatological indications, but it is FDA-approved only for systemic use, and varying results have been reported for topical formulations [23]. Given its physicochemical properties (lipophilicity and low molecular weight) the compound is expected to penetrate the outer layer of skin, the stratum corneum, well. However, the hydrophilic nature of the inner skin layers makes it more challenging for the drug to penetrate the inner skin layers to achieve its therapeutic effect. It remains unclear whether insufficient penetration or fast drainage with short exposure times limits its efficacy [24]. In either case, however, nanocarrier-based formulation may offer improvement. A 2% tofacitinib ointment has been used to test the anti-inflammatory efficacy of this drug when applied topically [25,26]. However, further studies are necessary to test tofacitinib’s long-term efficacy and safety for topical use [25]. Tofacitinib has also been encapsulated in amphiphilic squalenyl derivatives and tested as an anti-inflammatory agent in an ex vivo pig ear model and an in vivo mouse dermatitis model. This study demonstrated a sustained drug release in hair follicles but limited anti-inflammatory efficacy [27].

Since IL-6 is known to be important for various cutaneous inflammatory signaling pathways [28], the inhibition of the IL-6 pathway in inflamed tissue may offer a potent anti-inflammatory intervention by interrupting inflammation before dermal cells can be further hyperactivated. With the proven benefits of polymeric drug delivery systems [29,30], dendritic polymers such as dendritic polyglycerols, with varying morphologies and structures, have been studied conceptually in recent years as dermal drug delivery systems [31,32]. Most polyglycerol-based carriers are retained in the stratum corneum (SC) and may increase drug penetration into the viable skin layers over time [33]. Then, the distribution of polymeric carriers and their payload drug inside the skin are strongly dependent on the skin interaction with the carrier, drug characteristics, and drug release properties [34,35]. In recent years, immunosuppressive drugs such as tacrolimus and rapamycin were encapsulated in polyglycerol drug delivery systems and were tested as a treatment for inflammatory skin disease [36,37].

The past decade has seen the synthesis of dendritic polyglycerol sulfates (dPGS) bearing anti-inflammatory properties with efficient L or P selectin binding [38]. Importantly, previous studies have shown that dPGS binds to IL-6 [39] and significantly reduces neuroinflammation in an in vivo mouse model [40,41]. Moreover, our group has synthesized biodegradable polymers based on polyglycerol and polycaprolactone (dPG-PCL) and investigated their application as dermal drug delivery systems [33]. Building on the results of these studies, we aimed to investigate the anti-inflammatory efficacy of the JAK inhibitor tofacitinib encapsulated in dPGS-PCL nanocarriers as a combination therapy to treat inflammatory skin conditions in an ex vivo human skin model.

For this work, we synthesized dPGS-PCL through a 50 g scale copolymerization of glycidol and ε-caprolactone, followed by the sulfation of terminal hydroxyl groups. Analytical characterizations showed that the resulting polymer had a molecular weight of 54 kDa, an average size of 11 nm, and a −40 mV zeta potential. The high molecular weight and biodegradability of dPGS-PCL are advantageous for drug delivery. It has also been reported that sulfate groups have anti-inflammatory effects and can be used to combat inflammatory diseases [42]. We used liquid chromatography-tandem mass spectrometry (LC-MS/MS) to measure the loading capacity of dPGS-PCL with tofacitinib (11 w/w%), in-vitro drug release profiles in a pH of 7.4 and 5.0, and the ex vivo drug distribution in different skin layers. We also used the previously established ex vivo inflamed skin model to investigate the efficacy of the tofacitinib-loaded dPGS-PCL formulations in reducing the activation of the JAK-STAT pathway. Overall, our ex vivo and in vitro results suggest a role for tofacitinib-loaded dPGS-PCL as a safe and efficient dermal drug delivery system to treat chronic inflammatory conditions.

## 2. Results and Discussion

**Figure 1:**
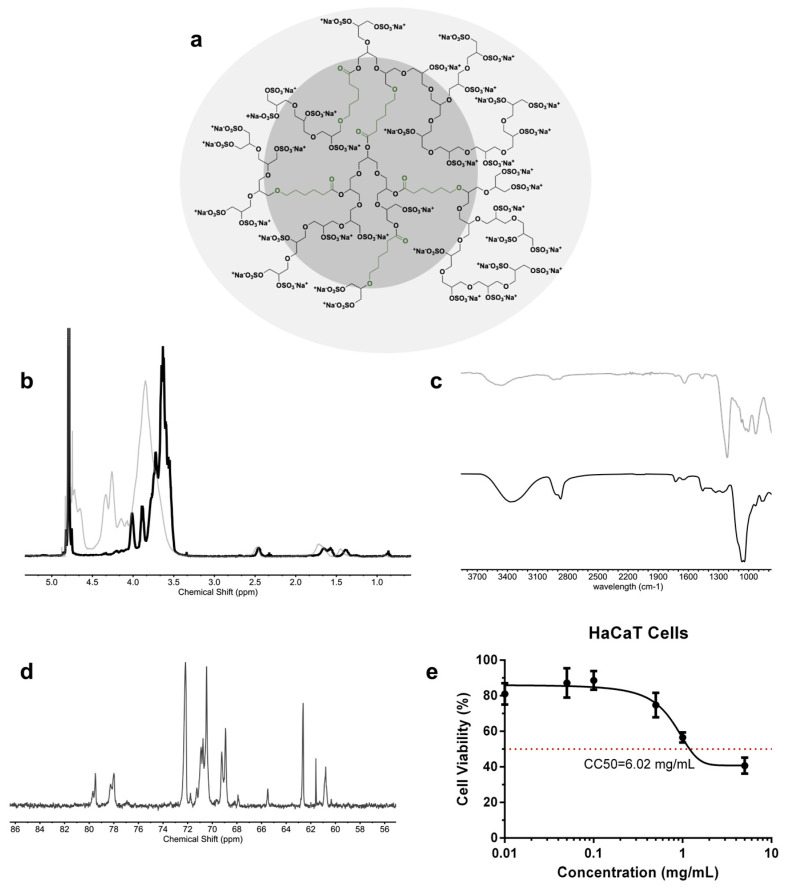
(**a**) Schematic structure of the dPGS-PCL, (**b**) ^1^H NMR of dPGS-PCL (gray) and dPG-PCL (black), (**c**) IR spectra of dPGS-PCL (black line) and dPG-PCL (gray), (**d**) ^13^C NMR inverse-gated in D_2_O NMR of dPGS-PCL, and (**e**) cell viability assay of dPGS-PCL for HaCaT cell line, red dot represents the half-maximum cytotoxicity concentration.

The one-step and gram-scale synthesis of dendritic polyglycerol with caprolactone segments on its backbone (dPG-PCL) was proven both scalable and reproducible [43]. The polymerization mechanism began with the catalytically and thermodynamically driven ring-opening polymerization of glycidol and ε-caprolactone in bulk. The second step consisted of the complete conversion of the hydroxyl groups into sulfate groups by reacting the product from step one with the sulfur oxide pyridine complex, resulting in a final product abbreviated as dPGS-PCL (Figure 1a). The successful sulfation of the hydroxyl groups was proved by ^1^H NMR and IR spectroscopy. In the ^1^H NMR spectra of dPGS-PCL (Figure 1b), the signals of methyl groups from the caprolactone segments and glycerol blocks are in the regions between 3 and 4.5 ppm, and 1 and 2.5 ppm, respectively. In the IR spectra of dPG-PCL and dPGS-PCL, the appearance of an absorbance band at 1650 cm^−1^ (Figure 1c), which was assigned to the carbonyl group, proved the incorporation of caprolactone segments into the structure of the polyglycerol.

The dPGS-PCL system was characterized using different analytical methods (Table 1). The degree of branching, calculated based on inverse-gated ^13^C NMR (Figure 1d), was 0.48. The molecular mass of the system before sulfating, the molecular weight of the system after sulfation, and the degree of sulfation, calculated from the elemental analysis, were 20 kDa, 54 kDa, and 95% respectively. Dendritic polyglycerols are highly water-soluble, and this property needed to be preserved even when including hydrophobic segments in its backbone. Therefore, the ratio of caprolactone incorporated in the backbone was calculated to be around 3.3% according to ^1^H NMR.

To study the biocompatibility of the dPGS-PCL, a cell viability assay was performed using the HaCaT cells monitored for 24 h of treatment using the CCK-8 test (Figure 1e). Over 24 h, dPGS-PCL showed no significant cytotoxicity up to a concentration of 5 mg/mL. The value of 6.02 mg/mL for the CC50, confirmed the high biocompatibility of the synthesized dPGS-PCL for dermal drug delivery applications.

**Figure 2:**
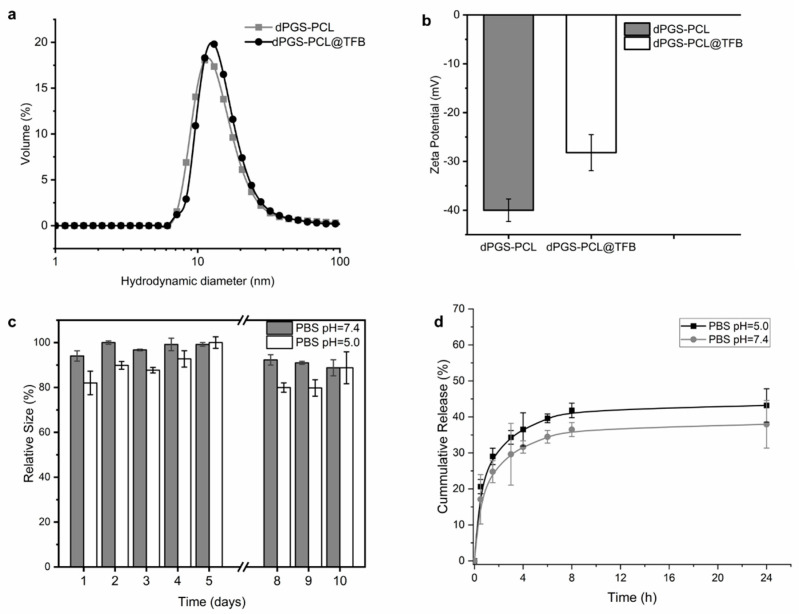
(**a**) Hydrodynamic size distribution of dPGS-PCL and dPGS-PCL@TFB in volume (%). Samples were prepared in PBS, pH = 7.4, c = 1 mg/mL. (**b**) Zeta potential of dPGS-PCL and dPGS-PCL@TFB. Samples were prepared in PB, pH = 7.4, c = 1 mg/mL. (**c**) Stability study of dPGS-PCL@TFB over 10 days in PBS, pH = 7.4 and pH = 5.0, c = 1 mg/mL. (**d**) In vitro release of tofacitinib from dPGS-PCL dermal drug delivery system at pH 5.0 and pH 7.4.

In this work, the pharmaceutical drugs tofacitinib citrate and sunitinib were encapsulated in dPGS-PCL and investigated for application as a dermal drug delivery system. The dPGS-PCL dermal drug delivery system, in an aqueous solution, was added dropwise to the vial containing the drug solution in methanol (c = 2 mg/mL) and stirred overnight in the dark. The therapeutic efficacy of tofacitinib citrate and sunitinib are limited by their low water solubility (47.9 µg/mL [27], and 4–8 µg/mL [44], respectively). Encapsulating these hydrophobic drugs in dPGS-PCL should increase their water solubility and therefore their therapeutic effectiveness. Sunitinib was used in this study for its fluorescence spectral properties as a hydrophobic dye. After purification with Sephadex^®^ G-25 in a PD-10 size exclusion column, the obtained loading capacities of the carrier systems were 11% and 7%, respectively. Both the empty carrier dPGS-PCL and the tofacitinib-loaded drug delivery system (dPGS-PCL@TFB) were further characterized by DLS to determine the hydrodynamic size (nm) and zeta potential (mV) for a 1 mg/mL solution in both PBS and PB; the size distribution was recorded in volume (%). The empty carrier has a size of 11 nm, compared to 14 nm for the loaded carrier (Figure 2a). As for zeta potential, the loaded carrier showed an increased zeta potential (−28.2 mV) compared to the empty carrier (−39.7 mV) (Figure 2b).

Tofacitinib citrate contains a tertiary amine that is protonated in water and interacts with the negatively charged sulfate groups of dPGS-PCL. The increase in zeta potential proves this assumption in practice, demonstrating that the interactions between tofacitinib and dPGS-PCL (dPGS-PCL@TFB) are electrostatic in nature. As for the stability of 1 mg/mL of dPGS-PCL@TFB in PBS solution at a pH of 7.4 and 5.0, the system proved relatively stable over a 10-day period. The two solutions were left on the bench and their hydrodynamic size was measured daily throughout this period. Over this period, the hydrodynamic size showed a smaller variation at a pH of 7.4 (<15%) compared to a pH of 5.0 (20%). We concluded that the solution was stable when stored in either media since the carrier size showed no noticeable variation (Figure 2c).

An in vitro release study of dPGS-PCL@TFB was performed at a pH of 5.0 and a pH of 7.4 using the dialysis bag method (Figure 2d). After 24 h, only 45% of the tofacitinib was released at a pH of 5.0, and 37% was released at a pH of 7.4. The increased release under acidic conditions was probably due to minor degradation of the dPGS-PCL dermal drug delivery system.

The skin serves as a physical and immunological barrier for the human body. Since its top layer, the SC, is the skin component most responsible for this function [45], topically applied drug delivery systems are typically retained in large amounts within the superficial layers of the SC. However, unhealthy skin conditions such as inflammation or infection degrade the integrity of the skin tissue’s barrier function, thereby increasing the tissue’s permeability [45]. A pilot experiment was therefore performed on intact and barrier-deficient skin models to evaluate the enhanced dermal drug delivery efficacy of dPGS-PCL for hydrophobic active agents within human skin. Three types of conditions were used: 30 times tape-stripped skin (TS) [46] to model mechanically damaged skin; serine protease (SP) pretreated skin (+SP) to model chemically damaged skin; and saline buffer pretreated skin (−SP) [37] to model an intact skin barrier. Skin samples were then treated with a low dose of sunitinib, ”as a model of hydrophobic dye”, loaded in dPGS-PCL (0.05 w/w%).

**Figure 3:**
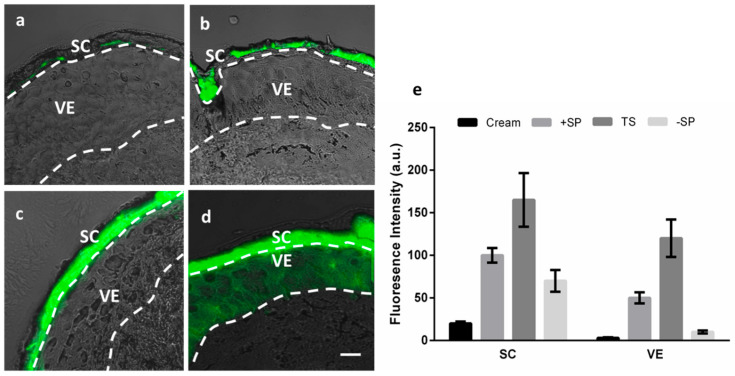
Representative overlay microscopy images (bright field and fluorescence) of human skin after 24 h topical application of (**a**) sunitinib dissolved in cream (intact skin), and dPGS-PCL@SUN (**b**) −SP skin model, (**c**) +SP skin model, and (**d**) 30 times tape stripped skin model. (**e**) Fluorescence intensity of sunitinib in different layers of human skin (*n* = 3, mean ± SD). SC: stratum corneum VE: viable epidermis +/− SP: with/without serine protease treatment, TS: tape-stripped skin. Scale bars correspond to 50 µm.

After a 24 h topical application of dPGS-PCL@SUN, the skin samples were cryo-sectioned and fluorescence images were taken (Figure 3a–d). As shown in Figure 3c,d, the sunitinib was highly distributed in the SC in both the TS and +SP skin samples due to the high permeability of skin in these models; even stronger fluorescence signals were observed in the viable epidermis of TS skin samples. Sunitinib, dissolved at the same dose (0.05 w/w%) as a commercially available base cream, served as a control (Figure 3a). In contrast to the +SP results, the sunitinib-treated skin of the −SP sample exhibited low fluorescence signals due to healthy skin’s lower permeability to external materials (Figure 3c). Image J software (Version 2.0) and picture analysis were also used to quantitatively measure the fluorescence intensity of sunitinib in different skin layers (Figure 3e). Clearly, dPGS-PCL enhanced the penetration of sunitinib in the SC and viable epidermis layers: 40-fold in the TS model, 16-fold in +SP, and 3-fold in −SP, all compared to the cream formulations.

**Figure 4:**
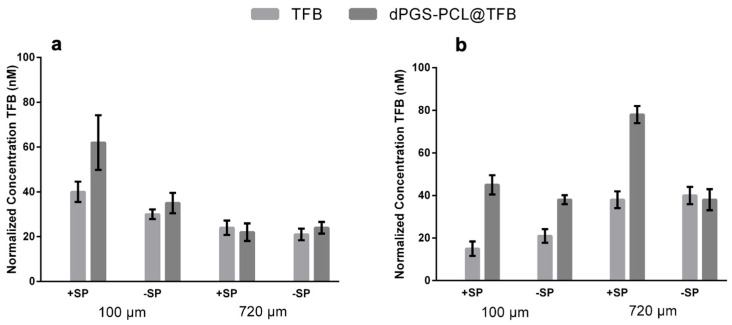
Drug concentrations in skin tissue after topical application of free TFB and dPGS-PCL@TFB followed by (**a**) 6 h or (**b**) 24 h incubation on ex vivo human skin. The drug concentration was determined in extracts of the epidermis (upper 100 µm) and the dermis (lowest 720 µm) of skin tissue. Three reported results were obtained from different donors treated (*n* = 3, mean ± SD). +/− SP: with/without serine protease treatment.

Our study also investigated the penetration of free tofacitinib and dPGS-PCL@TFB in both +SP and −SP human skin models over the time frames of 6 h and 24 h. Using the LC-MS/MS technique, drug penetration was measured quantitatively in different skin layers, at depths of 100 µm and 720 µm. As illustrated in Figure 4a,b, a constant penetration of tofacitinib was observed for both dPGS-PCL@TFB and free TFB in the deeper skin layer (720 µm) after 6 h of incubation. However, after 24 h of incubation (Figure 4b), a high amount of tofacitinib was detected in the deeper layers, especially for +SP skin samples. These results show that the dPGS-PCL dermal drug delivery system is able to penetrate the skin and deliver adequate drug throughout the incubation time of 24 h. However, after this interval, lower amounts of tofacitinib were detected in the skin samples treated with free TFB than in those treated with dPGS-PCL@TFB. In all experiments, at both the 6 h and 24 h time points, no drug was found in the medium beneath the skin or untreated control skin samples.

**Figure 5:**
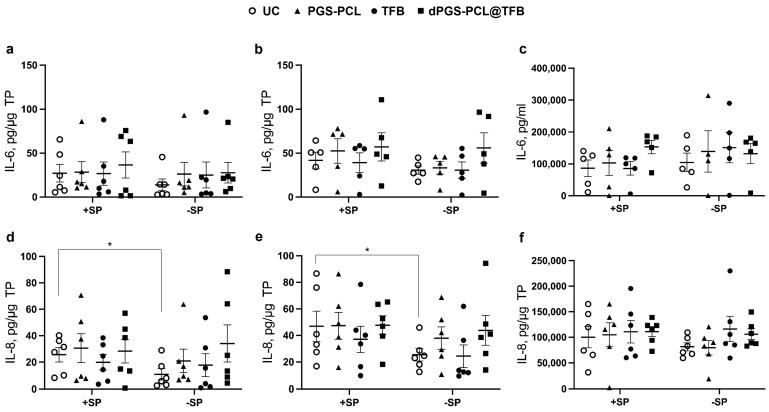
Effects of SP and TFB formulations on inflammatory markers IL-6 (**a**) and IL-8 (**b**). Skin samples were incubated for 16 h with (+SP) and without (−SP) topical trypsin (conc. 3 µg/cm^2^). Then, they were treated with the different TFB formulations for 24 h. Epidermis (**a**,**d**) and dermis (**b**,**e**) extracts as well as culture media (**c**,**f**) were analyzed for the content of the inflammatory cytokines using ELISA. * *p* < 0.05. No statistically significant differences were calculated between controls and treatments. Each symbol represents the value from each independent experiment. Lines represent the average and standard errors. UC = untreated control.

In a further step, we evaluated the effects of dPGS-PCL@TFB, dPGS-PCL, and free TFB on the JAK-STAT pathway using the developed ex vivo inflammatory skin model. Inflammation was induced by topical application of low concentrations of SP (3 µg/cm^2^). In previous studies, we showed that such a treatment induced a partial disruption of the SC, the release of inflammatory cytokines, and oxidative stress [39]. As in the previous studies, and also in this study, the inflammatory status of the skin samples was evidenced by monitoring the levels of IL-6 and IL-8 in the epidermis and dermis layers, and the culture medium (Figure 5). Variations between the samples were observed for both IL-6 and IL-8 concentrations. This probably reflects the inter-individual variability as well as the status of the sample after surgery and transportation. A baseline of IL-6 and IL-8 was observed in the −SP untreated control (UC) samples, indicating that, independently from specific treatments, background inflammatory processes were already activated in the skin explants. After SP treatment (+SP, UC), a slight increase of both cytokines in the epidermis and dermis, but not in the culture medium, was measured, with significant values for IL-8 in the epidermis and dermis. This indicates that the topical SP treatment can increase inflammation in the skin explants. In addition, it has been shown that the SP treatment induces skin barrier impairment with an increase in skin permeability. After the 24 h treatment with the different TFB formulations, no statistically significant differences, with respect to the respective controls, could be measured showing that such a short treatment is not enough to inhibit ongoing inflammatory processes. It was reported that JAK inhibitors, besides inhibiting the signal transduction of cytokines such as IL-6, can also reduce IL-6 levels [47,48]. However, these results were found in clinical studies after repeated drug administrations. As different cell populations release IL-6 as a result of different orchestrated intracellular pathways [49,50], it is improbable that a single drug application, as in our short-term experimental set-up, can reduce high IL-6 levels.

**Figure 6:**
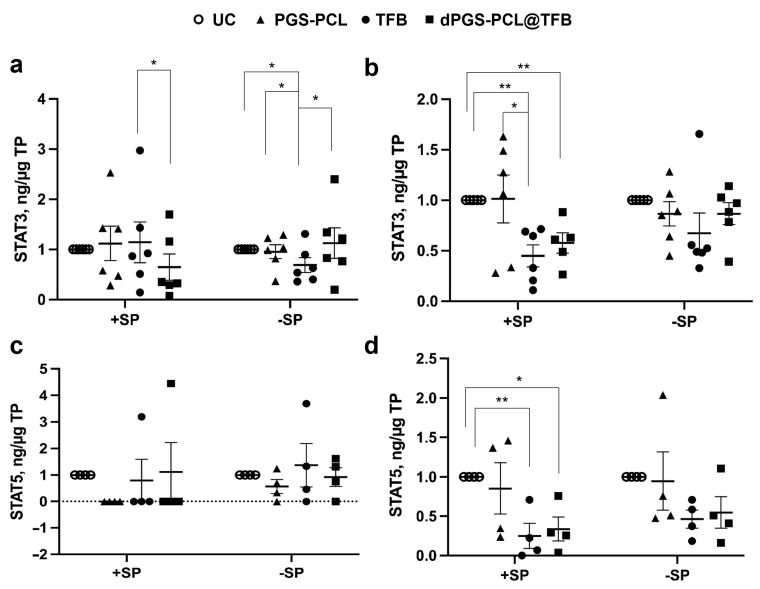
Effect of the TFB formulations on the JAK/STAT pathway. The amount of STA3 (**a**,**b**) and STAT5 (**c**,**d**) phosphorylation in the epidermis (**a**,**c**) and dermis (**b**,**d**) extracts was measured by ELISA. A reduction of phosphorylation correlated with the level of JAK inhibition. * *p* < 0.05; ** *p* < 0.01. Each symbol represents the value from each independent experiment. Lines represent the average and standard errors. UC = untreated control.

To monitor the effects of the TFB formulations on the JAK-STAT pathway, we measured the phosphorylation levels of STAT3 and STAT5, which reflect the activity of the JAK proteins. In general, we observed a reduction of STAT phosphorylation after the topical application of TFB formulations with or without a carrier. Interestingly, in the epidermis pre-treated with SP, the dPGS-PCK@TFB had a significantly lower level of STAT3 activation with respect to free TFB, while on the skin without SP pre-treatment, only TFB reduced STAT3 activation (Figure 6a).

Considering the deeper dermis layers, both the TFB-loaded carrier and free TFB significantly reduced STAT3 activation in SP-treated skin (+SP), while no significant differences could be measured in the skin with an intact barrier (−SP) (Figure 6b). As for STAT 5, a reduction of activity could be measured in almost all samples but only for skin with a disrupted SC (+SP) (Figure 6c). Similar results were found for the dermis of skin pre-treated with SP (Figure 6d). These results show that TFB, being a small drug, can penetrate the skin with a disrupted barrier and inhibit JAK-STAT activity. Nevertheless, a better effect in the epidermis of SP+ samples could be observed for the dPGS-PCL@TFB formulation. This is probably due to a better and faster drug delivery to the skin as seen from the drug release measurements after 6 h of incubation (Figure 4a).

These findings suggest that dPGS-PCL@TFB efficiently reduces the activation of STAT3 and STAT5 by increasing the penetration of the drug tofacitinib. The dPGS-PCL, deployed as a dermal drug delivery system containing JAK inhibitor drugs such as tofacitinib, may therefore represent a novel therapeutic method for treating various chronic inflammatory skin diseases such as psoriasis, atopic dermatitis, and alopecia areata.

## 3. Materials and Methods

### 3.1. Materials

Glycidol (96%) and ε-caprolactone (99%) were purchased from Acros Organics, (Morris Plains, NJ, USA) and were distilled prior to their use. Tin (II)-2-ethylhexanoate (Sn(Oct)_2_) and sulfur trioxide pyridine complex were purchased from Sigma-Aldrich (Hamburg, Germany). Tofacitinib citrate was purchased from Medchemexpress (Monmouth Junction, NJ, USA). Sunitinib was purchased from Selleckchem (Houston, TX, USA). A PD-10 Sephadex^TM^ G-25 Desalting Column was purchased from GE Healthcare (Stockholm, Sweden). For the purification of the polymer before sulfation, tangential flow filtration (TFF) was used with a 30 kDa molecular weight cut-off (MWCO) regenerated cellulose cassette (Merck, Darmstadt, Germany) in a cassette holder (Sartorius, Göttingen, Germany). A peristaltic pump (Gibson, Nashville, TN, USA) was used to circulate the solution into the system. Benzoylated cellulose dialysis membrane (Sigma-Aldrich, Hamburg, Germany, MWCO = 2 kDa) was used to purify the sulfated polymer.

### 3.2. Instruments

^1^H and ^13^C NMR spectra were recorded either on a Bruker AVANCE III 500 (Bruker Corporation, Billerica, MA, USA), a Jeol ECP 500 (JEOL GmbH, Freising, Germany), or a Bruker AVANCE III 700 (Bruker Corporation, Billerica, MA, USA). Deuterated water was used as the solvent, and the chemical shifts δ were reported in parts per million. Elemental analysis (EA) was performed with a VARIO EL (Elementar, Ronkonkoma, NY, USA). Gel permeation chromatography (GPC) measurements were performed in water using an Agilent 1100 (Agilent Technologies, Waldbronn, Germany). The instrument was equipped with a degasser, a pump, a UV detector, and an RI detector. For the separation of the samples, a precolumn and three columns with a particle size of 10 μm were used. Measurements were conducted at room temperature, injecting 50 μL of solution at a concentration of 6 mg/mL. The calibration standard was Pullulan, and the solvent was H_2_O with 0.1 M NaNO_3_. For Fourier transform infrared spectroscopy (FTIR), IR spectra were recorded on a Nicolet AVATAR 320 FT-IR 5 SXC (Thermo Fisher Scientific, Waltham, MA, USA) with a DTGS detector from 4000 to 650 cm^−1^. Sample measurement was performed by dropping a solution of the compound and letting the solvent evaporate for a few seconds. The particle size was determined via dynamic light scattering (DLS) measurements, which were carried out on a Zetasizer Ultra (Malvern Instruments Ltd., Malvern, UK) equipped with a He-Ne laser (nm). A backscattering mode was employed (detector angle: 173°). Samples were dissolved in a phosphate-buffered saline (PBS) solution, at a concentration of 1 mg/mL. UV-transparent disposable cuvettes (Plastibrand microcuvette) were used. For each measurement, 13 scans per sample were taken. UV-vis measurements were conducted on an Agilent Cary 8454 UV-visible spectrophotometer, using half-micro quartz cuvettes.

### 3.3. Synthesis of Biodegradable Dendritic Polyglycerol Sulfate (dPGS-PCL)

Dendritic poly(glycerol-co-caprolactone) copolymer sulfates (dPGS-PCL) were synthesized using a methodology previously established by our group [43]. In summary, a mixture of 52.5 mL of total monomers (comprising 45 mL of glycidol and 7.5 mL of ε-caprolactone) was introduced into a 1 L reactor vessel, equipped with a mechanical stirrer, at 100 °C under an inert atmosphere. The monomers were incrementally added via a syringe pump at a slow monomer addition (SMA) rate of 100 µL/min, and the catalyst was introduced in 0.6 mL batches every 2 h, totaling 2.4 mL. The reaction proceeded for 8 h until the polymer reached a highly viscous state, after which it was quenched with a water/ethanol mixture (90/10 *v/v*). The resulting polymer was then purified using a tangential flow filtration system with a 30 kDa MWCO membrane. Post-purification, the residual solvent was removed under reduced pressure and the product was subsequently lyophilized to yield a highly viscous solid. The next step involved the sulfation of the hydroxyl groups. The polymer from the prior step underwent a reaction with pyridine sulfur trioxide complex at 60 °C in an inert atmosphere overnight. This reaction was terminated using sodium hydroxide (NaOH), and the product was then dialyzed against a brine-to-water medium. Following solvent evaporation under reduced pressure, the final product was lyophilized and obtained as a salt compound.

### 3.4. Preparation of Drug-Loaded Drug Delivery Systems (DDS)

Two active pharmaceutical ingredients (API) were encapsulated in the drug delivery system, separately. The drug was encapsulated in the polymeric drug delivery system under a complete water-based protocol. The carrier was dissolved in water (c = 10 mg/mL) and added dropwise into a vial containing the drug for a total drug concentration of 2 mg/mL. The solution was then stirred overnight in the dark. The purification was performed by first centrifuging the solution at 4000 rpm for 5 min and passing it through a size-exclusion chromatography (SEC) column (DP-10 Sephadex G-25 Column). The resulting solution was again lyophilized to store the formulation as a solid compound. The loading capacity and the drug encapsulation efficiency were determined using UV-vis spectroscopy for DDS loaded with sunitinib (protocol page S1) and LC-MS/MS for tofacitinib-loaded DDS. The percentage loading capacity (*LC* %) was then calculated according to Equation (1).

(1) $$LC\left(\%\right)=\frac{amount\;of\;loaded\;drug\;(mg)}{total\;amount\;of\;obtained\;carrier-loaded\;drug\;(mg)}\times 100$$

For the further planned experiments, a gel formulation containing an end concentration of 0.05 (w/w%) and 0.5 (w/w%) of SUN and TFB per 1 mL aqueous solution was prepared, respectively. Hydroxyethylcellulose (HEC) was the reagent used to form gels. A total weight of 25 mg of HEC per 1 mL of the aqueous solution was used, and the mixture was then vortexed for 15 min to obtain the final gel formulation.

### 3.5. Stability of dPGS-PCL@TFB

The stability of dPGS-PCL@TFB loaded carriers was tested when kept at room temperature over a period of 10 days. The loaded dPGS-PCL was dissolved in phosphate-buffered saline (PBS) solution at a pH of 7.4 and 5.0 (c = 1 mg/mL). The variation in the hydrodynamic diameter was recorded through dynamic light scattering (DLS) every 24 h.

### 3.6. Cell Viability Assay of dPGS-PCL

The cytotoxicity of unloaded dPGS-PCL was evaluated using the Cell Counting Kit 8 (CCK-8) from Sigma Aldrich Chemie GmbH (Taufkirchen, Germany). Briefly, HaCaT cells (DSMZ no. ACC 771) were cultured in Roswell Park Memorial Institute (RPMI 1640) Medium from Fisher Scientific GmbH (Schwerte, Germany), supplemented with 10% fetal bovine serum (BioChrom KG, Berlin, Germany), 100 U/mL penicillin, and 100 μg/mL streptomycin. Cells were seeded in a 96-well plate at 4000 cells/well and incubated overnight at 37 °C in a 5% CO_2_ atmosphere. Subsequently, serial dilutions of the compounds were administered to the cells. Cells without treatment and those treated with 1% SDS served as the negative (untreated control) and positive controls, respectively. For the purpose of background subtraction, wells containing only the sample, but no cells, were also included. The cells were incubated for an additional 24 h at 37 °C, after which the CCK-8 solution was added. Absorbance readings were taken 3 h later at a wavelength of 450 nm and a reference wavelength of 650 nm using a Tecan plate reader (Infinitepro200, TECAN-reader Tecan Group Ltd., Männedorf, Switzerland). All measurements were conducted in triplicate and repeated three times. Cell viability was calculated by setting the absorbance of the non-treated control to 100% and that of the non-cell control to 0%, after adjusting for background using GraphPad Prism 6 software.

### 3.7. In Vitro Release Study from dPGS-PCL@TFB

The in vitro release profile of dPGS-PCL loaded with TFB (dPGS-PCL@TFB) was determined at a pH of 7.4 and 5.0 at 32 °C in PBS solution, as a control and to simulate skin conditions, respectively. The dPGS-PCL@TFB was dissolved in the respected medium (0.8 mL, c = 5 mg/mL) and inserted in the dialysis kit (Pur-A-lyzerTM Midi 6000, MWCO = 6–8 kDa). The dialysis kit was then placed in 15 mL of the acceptor solution, and the system was then placed in an incubator at 32 °C and shaken at 100 rpm. At different time points (t = 0, 0.5, 1, 1.5, 2, 6, 8, and 24 h), a 2 mL sample of the acceptor solution was taken, and an equal volume of fresh buffer was added. The samples were then lyophilized and redissolved in 40:60 water/acetonitrile with a final concentration of 500 nM internal standard [^13^C_3_,^15^N_1_]TFB. Then they were analyzed by LC-MS/MS to determine the amount of TFB released. Three experiments were conducted, and the results were expressed as the mean ± SD.

### 3.8. Ex Vivo Skin Penetration Study of Sunitinib

The skin penetration experiments of sunitinib-loaded dPGS-PCL (dPGS-PCL@SUN) (5 mg/mL) and sunitinib as a model of hydrophobic dye with close molecular weight to TFB (loading: 0.05 w/w%) were performed on freshly excised human skin [30,37]. The commercially available base cream containing (0.05 w/w%) sunitinib was used as a control. Human abdominal skin was obtained from cosmetic surgeries with the informed consent of healthy donors and ethical approval of the Charité−Universitätsmedizin Berlin (approval EA1/135/06, renewed in November 2019). Briefly, skin samples from three different donors were cut into pieces of 1.5 cm × 1.5 cm and fixed on the surface of Styrofoam blocks using needles. The prepared skin samples were kept in a box with wet towels to avoid skin tissue becoming dry. Subsequently, 40 μL of the sunitinib-loaded dPGS-PCL (according to the infinite dose approach) was applied to a 1 cm^2^ skin area. After 18 h of incubation at 37 °C, 5% CO_2_, and 95% humidity, unpenetrated materials from the skin surface were cleaned with cotton swabs. Then, the treated area of skin was cut with an 8 mm diameter Biospy-Punch. The skin samples were embedded in tissue freezing medium (Leica Microsystems, Wetzlar, Germany) and subsequently frozen in liquid nitrogen and stored at −20 °C. Sections of 5 μm thickness were obtained using a microtome (2800 Frigocut-N, Reichert-Jung, Heidelberg, Germany). Skin samples were subjected to confocal laser microscopy (LSM 700, Zeiss, Oberkochen, Germany). Pictures of at least 10 sections per donor were taken with a charge-coupled device (CCD) camera, always using the same settings. The mean fluorescence intensity (MFI) of sunitinib was analyzed using ImageJ software (version 1.47, National Institute of Health, Bethesda, MD, USA) for areas of SC and viable epidermis. The averages of at least 20 MFI values for each sample and controls from the three donors were calculated. Averages and standard deviations were plotted in diagrams using Microsoft Excel (Version 1808, Microsoft Corp., Redmond, WA, USA).

### 3.9. Ex Vivo Skin Penetration Studies of Tofacitinib Formulations

For the inflammatory ex vivo skin model, low doses of pig pancreas trypsin (Biochrom, Berlin, Germany), a serine protease (SP), were used to pre-treat the skin. To facilitate SP penetration into the SC and partially remove skin surface lipids, 50 µL of a 1:1 mix of methanol:chloroform (Merck KGaA, Darmstadt, Germany) was dropped onto a filter paper disc (SmartPractice, Phoenix, AZ, USA) that was placed on the top of the skin sample. After 1 min, the filter paper discs were removed and the skin pieces were transferred onto cell culture inserts (8 µm pore size membrane, BD Falcon™, Durham, NC, USA). The inserts were placed into six-well culture plates (BD Falcon™) and each well was filled with 2 mL of RPMI-1640 medium (PAA, Heidelberg, Germany) supplemented with 10% fetal calf serum (FCS, PAA, Heidelberg, Germany), 100 I.E./mL penicillin, and 100 g/mL streptomycin (Sigma-Aldrich, Hamburg, Germany). SP was applied on the top of the skin samples (20 µL of a 0.15 mg/mL solution, ie, 3 µg/cm^2^) leaving untreated margins of 0.5 cm to avoid the overflow of the applied solution. To the skin without SP treatment, 20 µL of 0.9% saline was applied topically. After 18 h of incubation at 37 °C, 5% CO_2_, and 95% humidity, SP and saline were removed using a cotton swab. Subsequently, 40 µL of the test materials were topically applied and samples were incubated for a further 24 h at the above-mentioned conditions. Thereafter, the skin surface was cleaned with cotton swabs and processed for the preparation of histological sections or tissue extracts.

### 3.10. Preparation of Skin Extracts

Horizontal sections of frozen skin (biopsy of 8 mm in diameter) were cut using a cryo-microtome (Frigocut 2800 N, Leica, Bensheim, Heppenheim, Germany). For the epidermis (the upper 100 µm), 5 sections of 20 µm in thickness were cut, whereas for the upper dermis (the lower 720 µm) 18 sections of 40 µm thickness were prepared. For the quantification of the drug and the IL-6 and IL-8 Enzyme-linked Immunosorbent Assay (ELISA), tissue slices were extracted by incubation of the sectioned tissue in 200 µL of acetonitrile (containing 200 nM internal standard [^13^C_3_,^15^N_1_]TFB) and 1 mL of cold extraction buffer (100 mM Tris-HCl; 150 mM NaCl; 1 mM EDTA; 1% Triton-X-100) in a Thermo mixer at 4 °C and 700 rpm, for 90 min, respectively. Samples were then sonicated (70 Hz, 200 Watt) for 10 min at 4 °C, vortexed, and centrifuged for 5 min at 380× *g* and 4 °C. The supernatants were stored at –20 °C and used for further analysis.

### 3.11. Quantification of Tofacitinib in In Vitro Release Assay Samples and Skin Extracts by Isotope-Dilution Liquid Chromatography Tandem-Mass Spectrometry

Analyses were conducted with a 1290 Infinity II HPLC coupled to an Ultivo Triple Quadrupole LC/MS system (both from Agilent Technologies, Waldbronn, Germany) interfaced with an electrospray ion source operating in the positive ion mode (ESI+). Chromatographic separation was carried out using a Zorbax Eclipse Plus C_18_ RRHD (2.1 mm × 50 mm, 1.8 μm) column. Ultra-purified water and acetonitrile (both acidified with 0.1% formic acid) were used as eluents A and B, respectively. Samples (0.5 to 1 µL) were injected into a mobile phase consisting of 90% of eluent A. TFB and its internal standard [^13^C_3_,^15^N_1_]TFB (Alsachim, Illkirch-Graffenstaden, France) were eluted from the column, which was maintained at 28 °C, with a 5 min linear gradient and a subsequent isocratic stage of 2 min with a 40:60 (v:v) eluent A/B at a flow rate of 0.3 mL/min. TFB and [^13^C_3_,^15^N_1_]TFB were co-eluted from the separation column at 2.2 min. The total run time for one analysis was 11.5 min, including re-equilibration of the LC system. The following ion source parameters were applied: drying gas temperature = 80 °C, drying gas flow = 12 L/min of nitrogen, sheath gas temperature = 400 °C, sheath gas flow = 11 L/min of nitrogen, nebulizer pressure = 60 psi, capillary voltage = 3500 V, and nozzle voltage = 500 V. The quantification of TFB in relation to the internal standard [^13^C_3_,^15^N_1_]TFB was carried out using the multiple reaction monitoring (MRM) approach. The following mass transitions were recorded (collision energies in parentheses): *m*/*z* 313.2 → 172.9 (40 eV), *m*/*z* 313.2 → 165.0 (20 eV), *m*/*z* 313.2 → 148.9 (28 eV), *m*/*z* 313.2 → 106.9 (72 eV), *m*/*z* 313.2 → 98.0 (32 eV) for TFB, and *m*/*z* 317.2 → 173.1 (40 eV), *m*/*z* 317.2 → 169.0 (20 eV), *m*/*z* 317.2 → 149.1 (32 eV), *m*/*z* 317.2 → 107.0 (72 eV), and *m*/*z* 317.2 → 98.0 (36 eV) for [^13^C_3_,^15^N_1_]TFB. The fragmentor voltage and dwell time were 130 V and 75 ms for each mass transition, respectively. The mass transitions *m*/*z* 313.2 → 148.9 (TFB) and *m*/*z* 317.2 → 149.1 ([^13^C_3_,^15^N_1_]TFB) served as quantifiers. Data analysis and quantification were performed using the MassHunter software (version 10.1, Agilent Technologies, Waldbronn, Germany).

### 3.12. Enzyme-Linked Immunosorbent Assay (ELISA)

IL-6 and IL-8 concentrations in skin extracts and culture media were measured using ELISA kits (Human IL-6 and IL-8 CytoSetTM (CHC1263, CHC1303) Invitrogen Corporation, Carlsbad, CA, USA) following the manufacturer instructions. The amounts of the cytokines in the skin extracts were normalized to the total protein content measured with a Pierce 660 nm Protein Assay (Thermo Fisher Scientific Inc., Rockford, IL, USA). Absorbance was measured with an EnSpire^®^ Multimode plate reader (Perkin Elmer, Akron, OH, USA).

The JAK inhibitory activity was measured by determining the levels of phosphorylated STAT3 and STAT5 proteins. The Human/Mouse Phospho-STAT3 (Y705) DuoSet IC ELISA was purchased from R&D System (Minneapolis, MN, USA) and the STAT5 alpha/beta (Phospho) [pY694/pY699] Human InstantOne™ ELISA Kit was purchased from Invitrogen Corporation, Carlsbad, CA, USA. For pSTAT5, the skin samples were extracted using the provided lysis buffer, whereas for pSTAT3 lysis buffer was prepared as follows: 1 mM EDTA, 0.5% Triton X-100, 5 mM NaF, 6 M Urea, 25 μg/mL Leupeptin, 25 μg/mL Pepstatin, 100 μM PMSF, 3.0 μg/mL Aprotinin, 2.5 mM Sodium Pyrophosphate, and 1 mM activated Sodium Orthovanadate in PBS, with a pH of 7.2–7.4.

### 3.13. Data Analysis and Statistics

The means, standard error of the means (SEMs), standard deviations (SDs), and statistics were calculated with Excel (Microsoft Corp., Redmond, WA, USA). For statistical analysis, a one-way ANOVA followed by a student’s *t*-test was used. Data were plotted using Prism GraphPad 6 (GraphPad Software, San Diego, CA, USA).

## 4. Conclusions

The sulfate functionalization of 95% of the terminal hydroxyl groups of polyglycerol polycaprolactone results in a water-soluble, highly functional, and biocompatible dPGS-PCL with outstanding physicochemical properties. The loading capacity of dPGS-PCL for tofacitinib was measured to be 11% *w*/*w*. This polymer’s low cytotoxicity, along with the enhanced dermal penetration of tofacitinib, was demonstrated in vitro and ex vivo, highlighting this system’s potential validity for future applications in dermal drug delivery. However, further in vivo validations will be required before these findings can be applied to future biomedical applications.

## Figures and Tables

**Table 1:** Physicochemical properties of dPGS-PCL.

[Gly]:[CL] ^a^	DB ^b^	[Gly]:[CL] ^c^	M_n_ before Sulfation (g/mol) ^d^	Đ ^e^	Mn after Sulfation (g/mol) ^f^	DS (%) ^g^
9:1	0.48	29:1	24,000	1.8	54,000	95

The values for ^a^ Feed [Gly]:[CL], ^b^ Degree of branching using inverse-gated ^13^C NMR in D_2_O, ^c^ [Gly]:[CL] in the product using ^1^H NMR in D_2_O, ^d^ M_n_ (g/mol) before sulfation and ^e^ polydispersity using GPC in water and ^f^ M_n_ after sulfating, and ^g^ Degree of sulfating (%) using elemental analysis were determined.

## Data Availability

Data is contained within the article.

## References

[1] Baumgarth N., Bevins C.L. (2007). Skin deep but complex. Nature.

[2] van Beelen A.J., Teunissen M.B., Kapsenberg M.L., de Jong E.C. (2007). Interleukin-17 in inflammatory skin disorders. Curr. Opin. Allergy Clin. Immunol..

[3] Lowes M.A., Bowcock A.M., Krueger J.G. (2007). Pathogenesis and therapy of psoriasis. Nature.

[4] Kapur S., Watson W., Carr S. (2018). Atopic dermatitis. Allergy Asthma Clin. Immunol..

[5] Darwin E., Hirt P.A., Fertig R., Doliner B., Delcanto G., Jimenez J.J. (2018). Alopecia Areata: Review of Epidemiology, Clinical Features, Pathogenesis, and New Treatment Options. Int. J. Trichol..

[6] Kortekaas Krohn I., Aerts J.L., Breckpot K., Goyvaerts C., Knol E., Van Wijk F., Gutermuth J. (2022). T-cell subsets in the skin and their role in inflammatory skin disorders. Allergy.

[7] Vesely M.D. (2020). Getting Under the Skin: Targeting Cutaneous Autoimmune Disease. Yale J. Biol. Med..

[8] Gudjonsson J.E., Kabashima K., Eyerich K. (2020). Mechanisms of skin autoimmunity: Cellular and soluble immune components of the skin. J. Allergy Clin. Immunol..

[9] Nestle F.O., Di Meglio P., Qin J.-Z., Nickoloff B.J. (2009). Skin immune sentinels in health and disease. Nat. Rev. Immunol..

[10] Tanaka T., Narazaki M., Kishimoto T. (2014). IL-6 in inflammation, immunity, and disease. Cold Spring Harb. Perspect. Biol..

[11] Johnson B.Z., Stevenson A.W., Prêle C.M., Fear M.W., Wood F.M. (2020). The Role of IL-6 in Skin Fibrosis and Cutaneous Wound Healing. Biomedicines.

[12] Haniffa M., Gunawan M., Jardine L. (2015). Human skin dendritic cells in health and disease. J. Dermatol. Sci..

[13] Howell M.D., Kuo F.I., Smith P.A. (2019). Targeting the Janus Kinase Family in Autoimmune Skin Diseases. Front. Immunol..

[14] Damsky W., King B.A. (2017). JAK inhibitors in dermatology: The promise of a new drug class. J. Am. Acad. Dermatol..

[15] Choy E.H., De Benedetti F., Takeuchi T., Hashizume M., John M.R., Kishimoto T. (2020). Translating IL-6 biology into effective treatments. Nat. Rev. Rheumatol..

[16] Zarrin A.A., Bao K., Lupardus P., Vucic D. (2021). Kinase inhibition in autoimmunity and inflammation. Nat. Rev. Drug Discov..

[17] Solimani F., Meier K., Ghoreschi K. (2019). Emerging Topical and Systemic JAK Inhibitors in Dermatology. Front. Immunol..

[18] Sakimoto T., Ishimori A. (2016). Anti-inflammatory effect of topical administration of tofacitinib on corneal inflammation. Exp. Eye Res..

[19] Singh R., Heron C.E., Ghamrawi R.I., Strowd L.C., Feldman S.R. (2020). Emerging Role of Janus Kinase Inhibitors for the Treatment of Atopic Dermatitis. Immunotargets Ther..

[20] Clark J.D., Flanagan M.E., Telliez J.-B. (2014). Discovery and Development of Janus Kinase (JAK) Inhibitors for Inflammatory Diseases. J. Med. Chem..

[21] Ciechanowicz P., Rakowska A., Sikora M., Rudnicka L. (2019). JAK-inhibitors in dermatology. Current evidence and future applications. J. Dermatol. Treat..

[22] Mustfa S.A., Maurizi E., McGrath J., Chiappini C. (2021). Nanomedicine Approaches to Negotiate Local Biobarriers for Topical Drug Delivery. Adv. Ther..

[23] Cárcamo-Martínez Á., Mallon B., Anjani Q.K., Domínguez-Robles J., Utomo E., Vora L.K., Tekko I.A., Larrañeta E., Donnelly R.F. (2021). Enhancing intradermal delivery of tofacitinib citrate: Comparison between powder-loaded hollow microneedle arrays and dissolving microneedle arrays. Int. J. Pharm..

[24] Gorantla S., Saha R.N., Singhvi G. (2021). Design of experiment-driven stability-indicating RP-HPLC method for the determination of tofacitinib in nanoparticles and skin matrix. Future J. Pharm. Sci..

[25] Bissonnette R., Papp K.A., Poulin Y., Gooderham M., Raman M., Mallbris L., Wang C., Purohit V., Mamolo C., Papacharalambous J. (2016). Topical tofacitinib for atopic dermatitis: A phase IIa randomized trial. Br. J. Dermatol..

[26] Szalus K., Trzeciak M., Nowicki R.J. (2020). JAK-STAT Inhibitors in Atopic Dermatitis from Pathogenesis to Clinical Trials Results. Microorganisms.

[27] Christmann R., Ho D.K., Wilzopolski J., Lee S., Koch M., Loretz B., Vogt T., Bäumer W., Schaefer U.F., Lehr C.M. (2020). Tofacitinib Loaded Squalenyl Nanoparticles for Targeted Follicular Delivery in Inflammatory Skin Diseases. Pharmaceutics.

[28] Nishimoto N., Kishimoto T. (2004). Inhibition of IL-6 for the treatment of inflammatory diseases. Curr. Opin. Pharmacol..

[29] Gupta S., Bansal R., Gupta S., Jindal N., Jindal A. (2013). Nanocarriers and nanoparticles for skin care and dermatological treatments. Indian Dermatol. Online J..

[30] Ahmadi V., Zabihi F., Rancan F., Staszak A.A., Nie C., Dimde M., Achazi K., Wiehe A., Vogt A., Haag R. (2021). Amphiphilic Co-polypeptides Self-Assembled into Spherical Nanoparticles for Dermal Drug Delivery. ACS Appl. Nano Mater..

[31] Gerecke C., Edlich A., Giulbudagian M., Schumacher F., Zhang N., Said A., Yealland G., Lohan S.B., Neumann F., Meinke M.C. (2017). Biocompatibility and characterization of polyglycerol-based thermoresponsive nanogels designed as novel drug-delivery systems and their intracellular localization in keratinocytes. Nanotoxicology.

[32] Hönzke S., Gerecke C., Elpelt A., Zhang N., Unbehauen M., Kral V., Fleige E., Paulus F., Haag R., Schäfer-Korting M. (2016). Tailored dendritic core-multishell nanocarriers for efficient dermal drug delivery: A systematic top-down approach from synthesis to preclinical testing. J. Control. Release.

[33] Mohammadifar E., Zabihi F., Tu Z., Hedtrich S., Nemati Kharat A., Adeli M., Haag R. (2017). One-pot and gram-scale synthesis of biodegradable polyglycerols under ambient conditions: Nanocarriers for intradermal drug delivery. Polym. Chem..

[34] Vogt A., Rancan F., Ahlberg S., Nazemi B., Choe C.S., Darvin M.E., Hadam S., Blume-Peytavi U., Loza K., Diendorf J. (2014). Interaction of dermatologically relevant nanoparticles with skin cells and skin. Beilstein J. Nanotechnol..

[35] Papakostas D., Rancan F., Sterry W., Blume-Peytavi U., Vogt A. (2011). Nanoparticles in dermatology. Arch. Dermatol. Res..

[36] Zabihi F., Graff P., Schumacher F., Kleuser B., Hedtrich S., Haag R. (2018). Synthesis of poly(lactide-co-glycerol) as a biodegradable and biocompatible polymer with high loading capacity for dermal drug delivery. Nanoscale.

[37] Rancan F., Guo X., Rajes K., Sidiropoulou P., Zabihi F., Hoffmann L., Hadam S., Blume-Peytavi U., Rühl E., Haag R. (2021). Topical Delivery of Rapamycin by Means of Microenvironment-Sensitive Core-Multi-Shell Nanocarriers: Assessment of Anti-Inflammatory Activity in an ex vivo Skin/T Cell Co-Culture Model. Int. J. Nanomed..

[38] Reimann S., Gröger D., Kühne C., Riese S.B., Dernedde J., Haag R. (2015). Shell Cleavable Dendritic Polyglycerol Sulfates Show High Anti-Inflammatory Properties by Inhibiting L-Selectin Binding and Complement Activation. Adv. Healthc. Mater..

[39] Ramsden L., Rider C.C. (1992). Selective and differential binding of interleukin (IL)-1 alpha, IL-1 beta, IL-2 and IL-6 to glycosaminoglycans. Eur. J. Immunol..

[40] Maysinger D., Lalancette-Hébert M., Ji J., Jabbour K., Dernedde J., Silberreis K., Haag R., Kriz J. (2019). Dendritic polyglycerols are modulators of microglia-astrocyte crosstalk. Future Neurol..

[41] Maysinger D., Ji J., Moquin A., Hossain S., Hancock M.A., Zhang I., Chang P.K.Y., Rigby M., Anthonisen M., Grütter P. (2018). Dendritic Polyglycerol Sulfates in the Prevention of Synaptic Loss and Mechanism of Action on Glia. ACS Chem. Neurosci..

[42] Kainthan R.K., Brooks D.E. (2007). In vivo biological evaluation of high molecular weight hyperbranched polyglycerols. Biomaterials.

[43] Cherri M., Ferraro M., Mohammadifar E., Quaas E., Achazi K., Ludwig K., Grötzinger C., Schirner M., Haag R. (2021). Biodegradable Dendritic Polyglycerol Sulfate for the Delivery and Tumor Accumulation of Cytostatic Anticancer Drugs. ACS Biomater. Sci. Eng..

[44] Huo M., Zhao Y., Satterlee A.B., Wang Y., Xu Y., Huang L. (2017). Tumor-targeted delivery of sunitinib base enhances vaccine therapy for advanced melanoma by remodeling the tumor microenvironment. J. Control. Release.

[45] Döge N., Avetisyan A., Hadam S., Pfannes E.K.B., Rancan F., Blume-Peytavi U., Vogt A. (2017). Assessment of skin barrier function and biochemical changes of ex vivo human skin in response to physical and chemical barrier disruption. Eur. J. Pharm. Biopharm..

[46] Zabihi F., Wieczorek S., Dimde M., Hedtrich S., Börner H.G., Haag R. (2016). Intradermal drug delivery by nanogel-peptide conjugates; specific and efficient transport of temoporfin. J. Control. Release.

[47] Valli A., Kuuliala K., Virtanen A., Kuuliala A., Palmroth M., Peltomaa R., Vidqvist K.-L., Leirisalo-Repo M., Silvennoinen O., Isomäki P. (2022). Tofacitinib treatment modulates the levels of several inflammation-related plasma proteins in rheumatoid arthritis and baseline levels of soluble biomarkers associate with the treatment response. Clin. Exp. Immunol..

[48] Li Y., Yuan L., Yang J., Lei Y., Zhang H., Xia L., Shen H., Lu J. (2019). Changes in Serum Cytokines May Predict Therapeutic Efficacy of Tofacitinib in Rheumatoid Arthritis. Mediat. Inflamm..

[49] Pattison M.J., Mackenzie K.F., Arthur J.S. (2012). Inhibition of JAKs in macrophages increases lipopolysaccharide-induced cytokine production by blocking IL-10-mediated feedback. J. Immunol..

[50] Szilveszter K.P., Németh T., Mócsai A. (2019). Tyrosine Kinases in Autoimmune and Inflammatory Skin Diseases. Front. Immunol..

